# Within-Host Dynamics of the Emergence of Tomato Yellow Leaf Curl Virus Recombinants

**DOI:** 10.1371/journal.pone.0058375

**Published:** 2013-03-05

**Authors:** Cica Urbino, Serafin Gutiérrez, Anna Antolik, Nabila Bouazza, Juliette Doumayrou, Martine Granier, Darren P. Martin, Michel Peterschmitt

**Affiliations:** 1 UMR BGPI CIRAD, Montpellier, France; 2 UMR Maladies Infectieuses et Vecteurs, Ecologie, Génétique, Evolution et Contrôle (CNRS 5290-IRD 224- UM1-UM2), Montpellier, France; 3 UMR BGPI, INRA, Montpellier, France; 4 Computational Biology Group, Institute of Infectious Diseases and Molecular Medicine, University of Cape Town, Observatory, South Africa; Virginia Tech, United States of America

## Abstract

*Tomato yellow leaf curl virus* (TYLCV) is a highly damaging begomovirus native to the Middle East. TYLCV has recently spread worldwide, recombining with other begomoviruses. Recent analysis of mixed infections between TYLCV and Tomato leaf curl Comoros begomovirus (ToLCKMV) has shown that, although natural selection preserves certain co-evolved intra-genomic interactions, numerous and diverse recombinants are produced at 120 days post-inoculation (dpi), and recombinant populations from different tomato plants are very divergent. Here, we investigate the population dynamics that lead to such patterns in tomato plants co-infected with TYLCV and ToLCKMV either by agro-inoculation or using the natural whitefly vector *Bemisia tabaci*. We monitored the frequency of parental and recombinant genotypes independently in 35 plants between 18 and 330 dpi and identified 177 recombinants isolated at different times. Recombinants were detected from 18 dpi and their frequency increased over time to reach about 50% at 150 dpi regardless of the inoculation method. The distribution of breakpoints detected on 96 fully sequenced recombinants was consistent with a continuous generation of new recombinants as well as random and deterministic effects in their maintenance. A severe population bottleneck of around 10 genomes was estimated during early systemic infection–a phenomenon that could account partially for the heterogeneity in recombinant patterns observed among plants. The detection of the same recombinant genome in six of the thirteen plants analysed beyond 30 dpi supported the influence of selection on observed recombination patterns. Moreover, a highly virulent recombinant genotype dominating virus populations within one plant has, apparently, the potential to be maintained in the natural population according to its infectivity, within-host accumulation, and transmission efficiency - all of which were similar or intermediate to those of the parent genotypes. Our results anticipate the outcomes of natural encounters between TYLCV and ToLCKMV.

## Introduction

Several plant viruses of the genus *Begomovirus*, family *Geminiviridae*, are well known for their economic importance [1]–[3] and their high propensity to recombine [4], [5]. Particularly notorious amongst these viruses is the highly invasive *Tomato yellow leaf curl virus* (TYLCV), which is transmitted–like all begomoviruses–in a circulative persistent manner by the whitefly *Bemisia tabaci* Genn. (*Hemiptera*: *Aleyrodidae*) [2]. During the past 30 years, TYLCV has spread from its native geographical range in the Eastern Mediterranean and the Middle East to numerous regions of the world following accidental introduction [6]. TYLCV has therefore only recently come into contact and had the opportunity to exchange genetic material with numerous begomovirus species that are indigenous to the environments it has invaded. It is therefore an excellent model with which to study the occurrence and impact of recombination during the geographical range expansion of viruses [6], [7]. Moreover, the small DNA genomes of begomoviruses can be manipulated with relative ease under laboratory conditions, facilitating their use for testing fundamental questions on viral recombination in multicellular hosts [8].

The outcome of recent contacts between invasive TYLCVs and indigenous begomoviruses has been particularly well described in the Western Mediterranean regions where TYLCV has been introduced independently at least three times from the Eastern Mediterranean over the past 30 years [6], [9], [10]. The meeting with *Tomato yellow leaf curl Sardinia virus* (TYLCSV)–a native of this region–has led to the emergence of various TYLCV-TYLCSV recombinants in Spain and Italy. Despite different strains of TYLCSV being endemic to Spain and Italy, and different TYLCV strains having been introduced independently to these countries, the recombinant genomes isolated from both countries exhibit similar mosaic structures: the virion sense gene side starting from the origin of replication has always been derived from the TYLCSV parent whereas the complementary sense gene side starting from the origin of replication and ending within the Rep gene or spanning all the complementary sense genes, has been derived from the TYLCV parent [11], [12]. Additionally, all the recombinants detected in an artificially reconstituted TYLCV-TYLCSV mixed infection [11], [13] resembled those found in natural infections. It is unknown to what degree either natural selection or variations in the basal recombination rates of different genome regions have contributed to the emergence of such “monomorphic” recombination patterns.

The recombination patterns generated in co-infections involving TYLCV and begomovirus species other than TYLCSV could be different. Recently, the recombination patterns arising during laboratory-constituted mixed infections of TYLCV and a begomovirus from the south west Indian Ocean (SWIO) islands, *Tomato leaf curl Comoros virus* (ToLCKMV, formerly named *Tomato leaf curl Mayotte virus* [14]), were analysed at 120 days post-inoculation (dpi) [15]. At this time point, recombinants were highly variable within and among plants and did not exhibit monomorphic recombination patterns. Despite this pattern variability, the distribution of the recombination breakpoints provided evidence of selection for recombinant patterns maintaining intra-genome interaction networks [15].

It is not yet clear why there is so much variability in the recombination patterns observed in co-infections with TYLCV and ToLCKMV compared to co-infections with TYLCV and TYLCSV. One possible explanation may come from a recent study on the impact of genome-wide random homologous recombination on viral fitness using TYLCV and ToLCKMV as parental genomes [8]. A large number of recombinants were generated by shuffling TYLCV and ToLCKMV genomes randomly, then selecting 47 of the resulting recombinants randomly for inoculation into tomato plants. All 47 recombinants were infectious and accumulated to levels similar or intermediate to those of the parental viruses, suggesting that most recombinants between TYLCV and ToLCKMV can be maintained in co-infected plants.

Two hypotheses might explain the variability of recombination patterns among the ten plants co-inoculated with TYLCV and ToLCKMV [15]. Firstly, if cell co-infection and recombination rates are high, then variability in recombination patterns among plants could be due to stochastic sampling from the recombinant population generated in each plant. Alternatively, cell co-infections and recombination could be rare and the few recombinants generated in each plant might differ among plants. Although the first hypothesis is supported by a previous report showing that, on average, 50% of the genomes were recombinant [15], stochastic effects were never investigated.

Our study has both an applied and a fundamental objective. The applied objective was to improve our ability to predict the outcome of contacts between TYLCV and ToLCKMV under natural conditions. Although these viruses presently have non-overlapping geographical ranges, they both occur on different islands of the South West Indian Ocean and are likely, given frequent past human-mediated movements of TYLCV, to come into contact at some time in the near future. A more fundamental objective was to detect drift effects during host colonization in co-infected plants and to estimate for the first time drift intensity for a single-stranded DNA virus. Questions related to both objectives were addressed with a set of 35 tomato plants co-infected by TYLCV and ToLCKMV and monitored for parental and recombinant genomes over time. Recombinant genomes were detected as early as 18 days post inoculation (dpi) and became predominant at 150 dpi. The parental genomes coexisted very differently with the recombinant genomes. The frequency of ToLCKMV was relatively constant overtime whereas the frequency of TYLCV decreased dramatically from 150 dpi onwards. Parental and recombinant frequencies over time were similar between both plants co-infected by agro-inoculation and plants co-infected using the natural whitefly vector, *B. tabaci*. According to several fitness components estimated for parental clones and a highly virulent recombinant detected multiple times in one of the co-inoculated plants, it was shown that this recombinant has the potential to emerge within natural populations. We observed different recombinant genotypes detectably appearing and disappearing at different time points within plants, suggesting that cell co-infection and recombination take place throughout infection. Based on the distribution of the recombination breakpoints over time within and between plants, drift as well as selection effects were detected. Using a competition test, we show that viral populations go through a severe population bottleneck during the early phase of systemic infection which may account for the high degrees of diversity in recombination patterns observed between different co-infected plants.

## Materials and Methods

### Plant Growth Conditions

Seeds of *Solanum lycopersicum* (L.) cv. ‘Monalbo’ (INRA) were grown in batches in containment growth chambers under 14 h light at 26±2°C, and 10 h dark at 24±2°C. Seven days after seeding, seedlings were transplanted to individual pots and were irrigated with 15∶10∶30 NPK+oligoelements. All experiments were conducted under these same environmental conditions.

### Parental Viral Genomes

Agro-infectious clones of TYLCV-Mild [Reunion: 2002] (accession n° AJ865337, here referred to as TYX) and ToLCKMV-[Mayotte:Dembeni:2003] (accession n° AJ865341, here referred to as TOX [14]) were used to co-infect tomato plants. These parental genomes were both used previously to generate a series of recombinants *in vitro* by DNA shuffling [8], and to study patterns of recombination arising in experimentally co-infected plants [15].

### Frequencies of Parental and Recombinant Genomes Over Time of Co-infection

**Figure 1:**
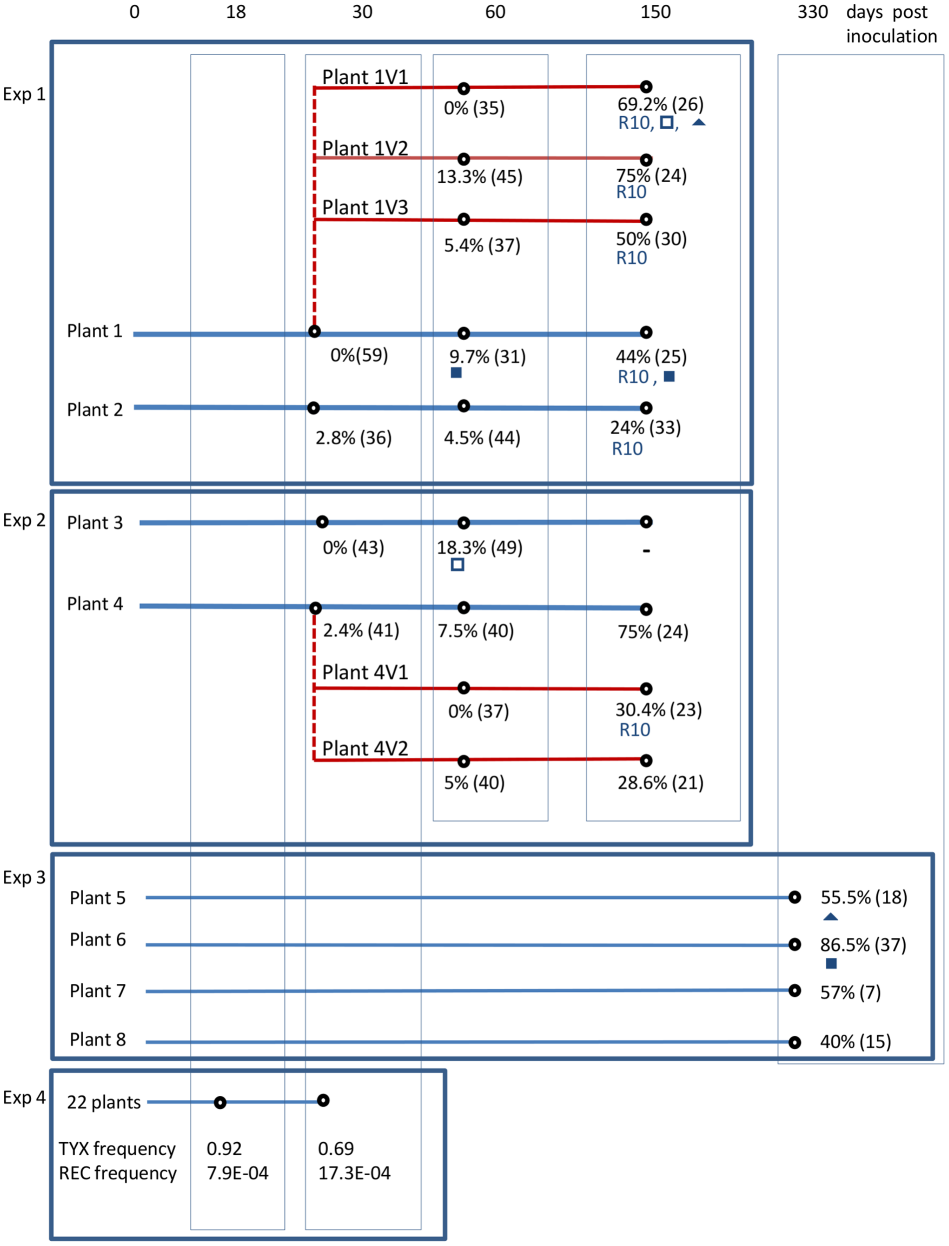
Origin of samples collected on tomato plants co-infected with TYX and TOX. Samples were collected from four independent experiments as indicated. Tomato plants were co-infected with *Tomato yellow leaf curl virus* (TYX) and *Tomato leaf curl Comoros viru*s;(TOX) either by agro-inoculation (blue lines) or by vector-inoculation (red lines). The vertical dotted red lines represent the vector transmission in which plant 1 and 4 were used as source plants. In Experiments 1–3, the percentage of recombinant genomes and the number of genomes analyzed (between brackets) were indicated at each sampling time. When the same recombinant genome was isolated from at least two distinct plants it is indicated by a symbol (▪, ▴, □) or by its name, (R10). (-) indicates no data obtained. In Experiment 4, the frequency of viral DNA of TYX and recombinant genomes (REC) in the viral population were estimated at 18 and 30 days post inoculation (dpi) by real time PCR. The frequency was estimated from the five plants which were detected positive for recombinants at 18 dpi and from the 22 plants detected positive at 30 dpi.

Four experiments (Figure 1) were conducted to estimate the frequencies of parental and recombinant genotypes during the co-infection of tomato plants. Genotype frequencies were estimated on samples collected between 30 and 150 dpi in Experiments 1 and 2, at 330 dpi in Experiment 3, and between 18 and 30 dpi in Experiment 4.

#### Agro-inoculation of tomato plants

*Agrobacterium tumefaciens* cultures harbouring infectious viral clones were grown for about 26 h at 28°C in a liquid LB medium containing kanamycin (50 µg/ml) and gentamycin (20 µg/ml), to an optical density (OD) of between 3 and 5. To co-infect the two parental viral clones at the same concentrations, agro-infectious cultures were adjusted to identical ODs by dilution with LB medium, mixed in equal volumes and finally concentrated ten-fold by centrifugation (20 min at 1,000 g) and resuspended in LB medium. Cotyledon leaves of 14-day old tomato plants of the susceptible cultivar Monalbo were agro-infiltrated as described in [8]. Plants infected with both parental viruses were identified before 31 dpi with Taqman quantitative PCR assays specifically designed to detect TYX and TOX (see below).

#### Vector-inoculation

For experiments 1 and 2, two plants with TYX:TOX ratios of viral accumulation closest to 1 were kept for the experiment and one of these was used as a source plant for vector transmission. *B. tabaci* individuals were collected from a rearing population of the putative species Med (Mediterranean, previously reported as the Q biotype) and originating from the Roussillon region of Southern France. Approximately 400 freshly emerged insects were given an acquisition access period (AAP) of 48 h on a tomato plant 29 days post co-inoculation with TYX and TOX (plant 1 in Exp 1 and plant 4 in Exp 2; Figure 1). At the end of the AAP, females were selected after observation under a stereo microscope and transferred individually to 14-day old tomato plants (91 test plants in Exp 1 and 53 in Exp 2) for a 48-h inoculation access period (IAP). Co-infected plants were identified by Taqman real-time PCR (described below) 28 days after vector transmission.

#### Collection and analysis of plant samples in experiments 1–3

In experiments 1 and 2, plant samples were collected from four agro-inoculated plants (plants 1–4) and from five vector-inoculated plants (plants 1V1, 1V2, 1V3 in Exp 1 and 4V1, 4V2 in Exp 2) (Figure 1). Each of the four agro-inoculated tomato plants was sampled at 30, 60 and 150 dpi by collecting 200 mg of young parts per plants including leaf stem and apex (Figure 1). The five plants that were co-infected by an individual whitefly were similarly sampled 30 and 120 days after vector transmission. Since the mixed viral population acquired by the whitefly vectors had already spent 30 days on the source plant, the viral populations sampled at 30 and 120 days after vector transmission were thus considered to be 60- and 150-days old, respectively (Figure 1). In experiment 3, four co-infected plants (5, 6, 7 and 8), were sampled at 330 dpi only (Figure 1).

**Figure 2:**
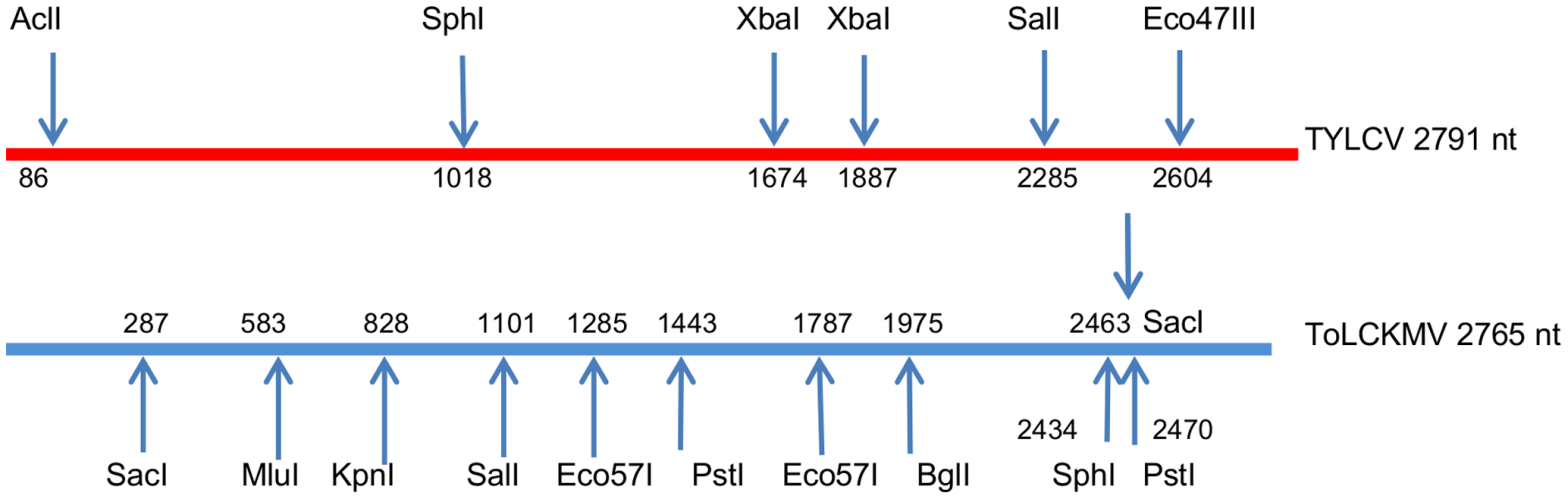
Nucleotide positions in *Tomato yellow leaf curl virus* (TYX) and *Tomato leaf curl Comoros virus* genomes of restriction endonuclease restriction sites. The origin of virion strand replication is at position 1 for *Tomato yellow leaf curl vi*rus (TYX) and *Tomato leaf curl Comoros virus* (TOX).

Total DNA was extracted from the plant samples using a Mini DNeasy plant DNA extraction kit (Qiagen, Courtaboeuf, France) and used as template for the amplification of circular DNA molecules using a TempliPhi Amplification kit (GE Healthcare, U.K.) according to the manufacturer’s instructions. This method has been shown to not generate artifacts resembling recombinants produced in coinfected plants [13]. Full-length 2.8-kb begomovirus genomes were cloned using a unique restriction site (*Xho*I) and purified from agarose gels following electrophoresis (SV Wizard Gel kit, Promega, Madison, USA). The *Xho*I-restricted genomes were ligated to the *Xho*I site of the vector pCambia 0380-TB [8], in which they can be tested directly for infectivity by agro-inoculation as described previously [16]. Bacterial clones shown to contain recombinant plasmids with full genome-sized inserts were selected randomly from plates for restriction fragment length polymorphism (RFLP) analysis with 11 restriction enzymes that digest specifically 17 restriction sites distributed throughout the two parental viral genomes (Figure 2): 31 to 59 clones were tested for each sample collected at 30 and 60 dpi; 21 to 45 clones for those collected at 150; and 7 to 37 for those collected at 330 dpi. Clones for which restriction patterns differed in TYX and TOX profiles were considered as recombinants. All the genomes of the 30 dpi and 60 dpi samples identified to be recombinant by RFLP were sequenced (Cogenics Mylan, France). In the later samplings we sequenced at least one representative recombinant of each distinct RFLP pattern per sample (54 clones sequenced out of the 101 detected at 150 dpi, and 16 clones sequenced out of the 55 detected at 330 dpi). Samples collected at 330 dpi from plants 7 and 8 were analysed only with RFLP. Sequence data were analyzed using the computer program DNAMAN (version 7.0, Lynnon BioSoft, Quebec Canada). The frequency of parental or recombinant genomes was calculated as the number of such identified clones divided by the number of clones analyzed per sample.

The frequencies of parental and recombinant genomes observed at different dates of sampling were analysed using a generalized linear mixed model GLMM (glmer function in R library lme4, [17]) with a binomial distribution. The time and the inoculation method were specified as fixed effects and the infected sampled plants as a random effect to account for repeated measures of the same plant over time.

#### Collection and analysis of plant samples in experiment 4

A total of 22 TYX-TOX co-infected plants were sampled at 18 and 30 dpi (Figure 1). Each plant sample consisted of a total of five 4-mm-diameter leaf disks collected from the five youngest leaves, one disc per leaf and stored at –80°C. DNA extraction was performed with the extraction protocol of [18] modified as follows: leaf tissue was ground in 400 µL extraction buffer (100 mM Tris-HCl, pH 8.0, 50 mM EDTA, 500 mM NaCl, 0.8% w/v Na_2_SO_3_, and 100 µg/ml RNase), incubated at 65°C for 10 min and centrifuged (13,000 rpm, 10 min). After addition of 1 volume cold iso-propanol to the supernatant, nucleic acids were recovered (12,000 rpm, 20 min), resuspended in 50 µL sterile distilled water and either processed further immediately or stored at −20°C.

The proportion of recombinants in the total viral population was determined with SYBR Green real-time PCR. Primers TYO 2164+ and TYO 2339– (formerly called TY 2164+ and TY 2339– [8]), were designed to bind to genome regions that were identical between TYX and TOX (Table 1). As they could detect parental as well as recombinant genomes generated from these parental viruses, they were used to quantify the total viral genomes in co-infected plants. They were tested in a 10 µL reaction mix comprising the 2× LightCycler® 480 SYBR Green I Master kit (Roche, Germany), each primer at the optimised concentration (Table 1), and 2 µL of a 1/100 dilution of the DNA template. The amplification reactions were run in 384-well optical plates in Roche LightCycler System (Roche, Germany). Two replicates were amplified per sample. Cycling parameters were 95°C for 10 min followed by 40 cycles of 15 s at 95°C, 20 s at 66°C and 15 s at 72°C. The number of viral genome copies was estimated using a standard curve obtained with a serial tenfold dilution of the plasmid containing the TYX genome (1.14×10^3^–1.14×10^9^ copies) in a 1/100 dilution of a mock-DNA extract from healthy tomato plants. The corresponding standard curve had high correlation coefficients (R^2^>0.99), and calculated PCR efficiencies ranged from 98% to 102%. All PCR fluorescence data were analyzed using the 2nd derivative max function of the LightCycler® 480 Software and LinReg computer program [19]. The starting concentration of the target, called N0, is expressed in arbitrary fluorescence units per sample. N0 is calculated from the values of the Fluorescence threshold (Ft), which is the fixed amount of fluorescence on each plate, E_mean_, the PCR efficiency calculated for each well, and Ct, the fractional number of cycles needed to reach the fluorescence threshold:
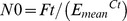



**Table 1:** Description of primers and probes used in real time PCR.

Primer	Sequence 5′-3′	Final concentration
TYO 2164+	CTAAGAGCCTCTGACTTACTGC	200 nM
TYO 2339-	AACATTCAGGGAGCTAAATCCAG	200 nM
TYX 2616+	GAGTACCGATTGACCAAGATTTTTACACTT	200 nM
TYX 44-	GTGACGTCTGTGGAACCCT	200 nM
TOX 2648+	TGGCAATCGGGGGAACTGGGGGGCA	200 nM
TOX 78-	CTAAGCTTTGACGCGCGATTCTT	200 nM
TYX 2575+	CAATTTATTTGGAAGCGCTTAGGAGGAGCCATA	200 nM
**Taqman system for Tyx**		
TYX 2591+	GCTTAGGAGGAGCCATAT	300 nM
TYX 2661-		300 nM
Probe TYX 2636–2613	AATCTTGGTCAATCGGTACTCATT	250 nM
**Taqman system for Tox**		
TOX 2561+	GGCTTTGATGCTGAAACGA	300 nM
TOX 2644-	CCCGATTGCCATAGACTTTG	300 nM
Probe TOX 2581–2605	CGGCTCTCGGCATCTTCTATGTCGT	250 nM

Primers (TYX 2575+, TOX 78–, Table 1) amplified all recombinant genomes (Rec) that harboured a breakpoint in the intergenic region, with a sequence originating from TYX on the left part and a sequence originating from TOX on the right part of the origin of replication. As this region is a known recombination hot-spot [4], and also because more than half of the recombinant profiles detected in both our study and that of [15] presented this profile, these primers were used to estimate the proportion of recombinant genomes in co-infected plants. The amplification reactions were run and analysed as described above in 384-well optical plates in Roche LightCycler System (Roche, Germany). Cycling parameters were 95°C for 10 min followed by 40 cycles of 10 s at 95°C, 10 s at 60°C, and 15 s at 72°C. Relative frequencies (Rf) of recombinant genomes were calculated in each sample from the N0 values obtained with primers TYX 2575+, TOX 78– (N0_Rec_), and primers TYO 2164+ and TYO 2339– (N0_Total_) using the following formula:
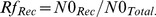



Considering the range of Ct obtained when testing healthy tomato plants with recombinant primers [31.4, 40.0], the positive detection threshold was fixed at 30 Ct.

Specific detection and relative accumulation of TYX and TOX was performed with Taqman real-time PCR. Primers and probes (Table 1) were designed using Beacon Designer (V3.0, Premier Biosoft International, Palo Alto, CA, USA). The 5′- and 3′-ends of the probes were labelled with the fluorescent dyes FAM (6-carboxyfluorescein, excitation wavelength = 494 nm, emission wavelength = 521 nm) and BHQ-1 (Black Hole dark quencher, quenching range 480–580), respectively. Two different systems of primers and probes were designed for detecting TYX and TOX respectively. Cycling parameters were 95°C for 10 min followed by 40 cycles of 10 s at 95°C, 15 s at 55°C (TYX) or 52°C (TOX) and 20 s at 72°C; 5 µl of each 1/100 diluted DNA sample were tested in 25 µL reaction mixes comprising the 2× qPCR MasterMix Plus Low ROX (Eurogentec, Seraing, Belgium), each primer at the optimised concentration (Table 1) and 5 µL of a 1/100 dilution of the DNA template. The amplification reactions were run on 96-well optical plates and the M×3005P QPCR System (Agilent Technologies, La Jolla, CA, USA). Two replicates were amplified per sample. Data obtained by real-time PCR were analyzed using MxProv4 (Agilent Technologies, La Jolla, CA, USA).

The number of DNA copies of TYX and TOX was assessed with standard curves as described above with a serial tenfold dilution of the plasmids containing each of the viral genomes. The corresponding standard curves had high correlation coefficients (R^2^>0.99), and calculated PCR efficiencies ranged from 98% to 100%. Results were expressed as the ratio of the numbers of copies of TYX:TOX.

### Infectivity of Six Recombinant Genomes in Tomato Plants

Six recombinant clones (R4, R10, R5–20, R6–91, R6–16 and R6–22), along with TYX and TOX were agro-inoculated to 11 to 20 tomato plants each, as described above. At 30 dpi, leaf discs were sampled from each plant and tested for virus infection by SYBR Green real time PCR with primers TYO 2164+ and TYO 2339–, as described above.

### Estimation of Fitness Components

#### Agro-inoculation

The infectivity and within-host accumulation of the most severe recombinant genotype (R4) was estimated in four independent tests (tests 1 to 4) after co-inoculation with the two parental viruses. A total of 60 (test 2 and 3) or 90 (test 1 and 4) tomato plants were co-inoculated as described above with an equal mix of an *A. tumefaciens* culture of TYX, TOX and R4. A total of 35 to 60 tomato plants were co-inoculated with an equal mix of TYX and TOX cultures as controls. As the infectivity of TOX was generally very low [8], agroinfectious clones were cultured in NZY+ medium containing Acetosyringone 150 µM and MgCl2 10 mM, instead of LB medium in test 3, as this media was expected to globally improve infectivity.

#### Infectivity and viral load

At 18 and 30 dpi, five leaf discs were collected from each plant as described above. Specific detection of each viral genome was achieved with SYBR Green real-time PCR tests using primers (Table 1) designed to specifically distinguish between TYX (TYX 2616+, TYX 44–), TOX (TOX 2648+, TOX 78–) and R4 (TYX 2575+, TOX 78–). The mix was prepared as described above with the LightCycler® 480 SYBR Green I Master kit (Roche) and two replicates were amplified per sample. Cycling parameters were 95°C for 10 min followed by 40 cycles of 10 s at 95°C, 10 s at 60°C (TYX and REC), or 65°C (TOX) and 15 s at 72°C. All PCR fluorescence data were analyzed as described above for SYBR Green real-time PCR tests.

To evaluate competition between R4 and parental clones, viral loads were estimated from No values (see above) at 18 dpi and 30 dpi and compared with an ANOVA test after a logarithmic transformation.

The independence of infectivity between co-inoculated viruses was determined from the number of plants detected for each possible combination. Fisher’s exact test was used to analyse the four combinations obtained from the TYX-TOX inoculation tests. For the TYX-TOX-R4 inoculation tests, the G2 statistic was used for the eight combinations resulting from the inoculation and its significance was obtained using permutation tests [20]. The test is obtained by 1,000 permutations of the table with constant margins, for each of which G2 is calculated. Statistical analyses were carried out using R 2.8.1 software [21].

#### Efficiency of transmission by the vector B. tabaci

The efficiency of virus transmission by whiteflies was assessed by determining the percentage of infected plants following inoculation with a single whitefly vector per plant. For each viral clone, whiteflies were given a 48 h AAP on three individually caged infected tomato plants. Female whiteflies collected on each source plant were then caged individually on 30 (for TYX) or 40 (for TOX and R4) 14-day old tomato plants for a 5-day IAP. At 30 dpi, leaf discs were collected from each plant as described above. Infected plants were identified by SYBR Green real-time PCR with primers TYO 2164+ and TYO 2339–, as described above. The transmission efficiencies were analysed with a generalized linear model (GLM) and a hierarchical GLM model with a binomial distribution (JMP 10, SAS Institute Inc, Cary, North Carolina, USA). The Fisher’s exact test was used to compare the transmission efficiency of R4 with that of parental clones (R 2.8.1 software [21]).

### Population Bottleneck Size Estimation

The relative frequencies of TYLCV and R4 were determined at 18 and 30 dpi from the competition tests described above and were used to estimate the size of the within-host population bottleneck. Relative frequencies of R4 (*Rf R4*) were calculated for each sample from the N0 values obtained with each couple of primers using the formula:
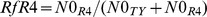



The method used is based on the comparison of genetic variability between two populations: an initial population and a population founded by individuals from the initial population [22]. In our experiment, the sampling procedure is expected to yield a population representative of the circulating population at each sampling date since it involved collecting samples from each of the five leaves of the apex. Hence, we can compare the “initial” population at 18 dpi with the “resulting” population at 30 dpi. The method also requires that the population is composed of equally-fit genotypes, which means that the relative frequency of TYX and R4 should not differ significantly between 18 and 30 dpi. R4 relative frequencies were analyzed using MANOVA between 18 and 30 dpi (repeated measures; JMP 10, SAS Institute Inc, USA). Any change in the frequencies of equally-fit phenotypes between the two populations is then only attributable to stochastic processes during host colonization. Thus the population bottleneck endured by the viral population within the plant between the two dates is estimated by comparing the variance in the relative frequencies of TYLCV and R4 among plants.

The size of the population bottleneck can be derived from the following formula where N is the population bottleneck size, p is the mean frequency of R4 at 18 dpi, and Var18 and Var30 are the variances in R4 frequency at 18 and 30 dpi, respectively.
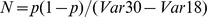



We used a bootstrap re-sampling technique with replacement to obtain 1,000 estimates of N. These estimates were then used to create a distribution of N and to estimate its median value and 95% confidence intervals. When bootstrapped datasets yielded a negative value of N because their initial variance was larger than their final variance, these N values were discarded from the distribution as reported in [22]. The median value of the distribution was considered representative of the estimate of the bottleneck size as shown in [22].

## Results

### Frequency of Recombinant Genomes Over Time in Agro-inoculated and Vector-inoculated Plants

**Figure 3:**
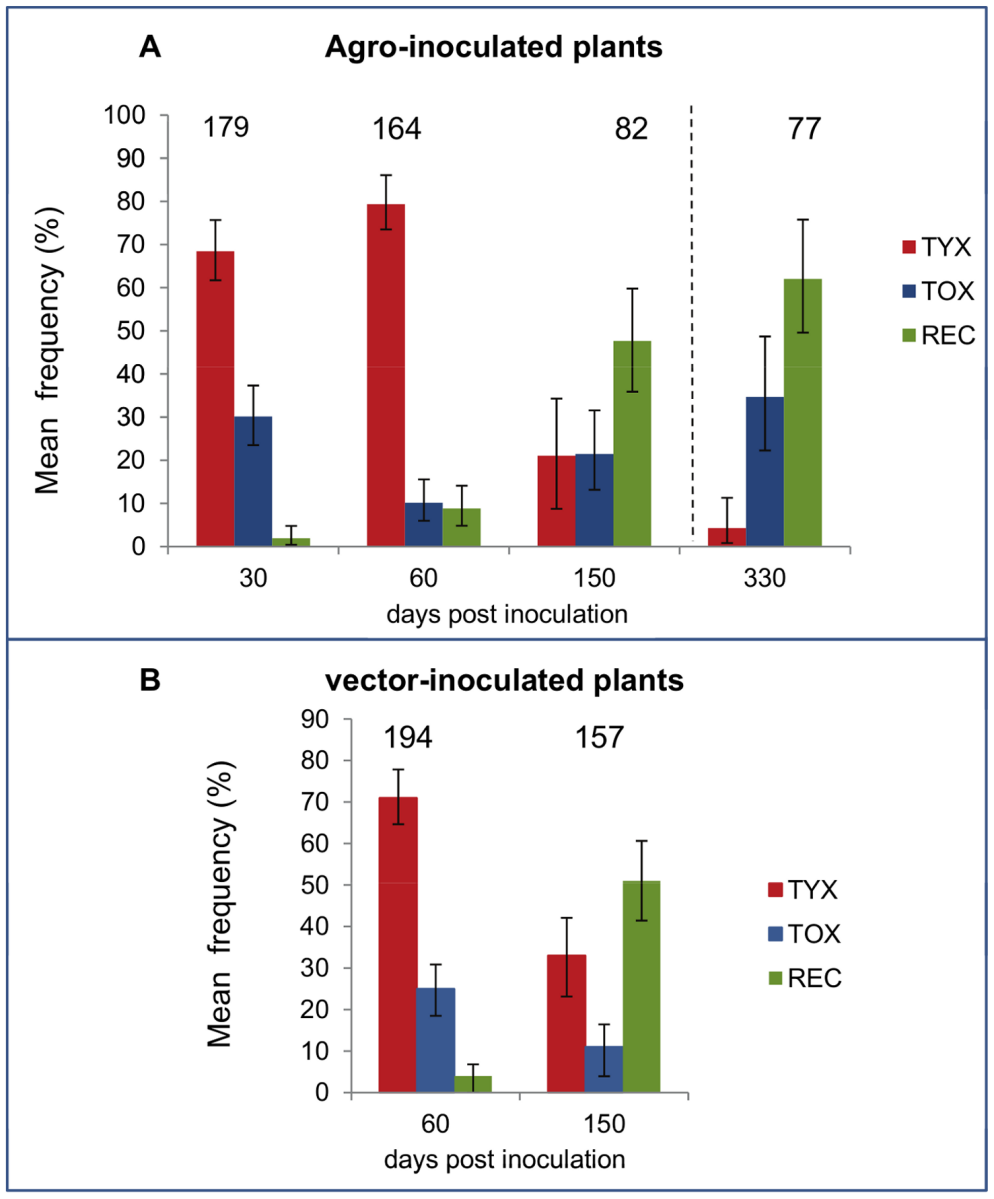
Frequency of TYX, TOX and recombinant (REC) genomes in co-infected tomato plants. Following co-infection of plants with *Tomato yellow leaf curl virus* (TYX) and *Tomato leaf curl Comoros virus* (TOX), the frequency of parental and recombinant genomes was monitored within plants at different days post-inoculation (dpi). **A**) Frequencies in four tomato plants co-infected by agro-inoculation and sampled at 30, 60 and 150 days post inoculation (dpi). The 330 dpi results were from four plants of a separate experiment as indicated in Figure 1. **B**) Frequencies in five tomato plants co-infected by vector-inoculation and sampled at 60 and 150 days after the agro-inoculation of the source plants used for vector transmission as indicated in Figure 1. The numbers at the top of the graphs indicate the total number of genomes analyzed by restriction analysis. The error bars are 95% confidence intervals of the mean frequency.

The frequency of parental and recombinant genomes was determined using restriction mapping of full length genomes isolated at different sampling time-points from 13 tomato plants co-infected with TYX and TOX (Figure 1). At 30 dpi, the earliest sampling point, 1.1% recombinant genomes were detected on average from four agro-inoculated plants (Figure 3A). The proportion of recombinants increased significantly over time to reach 47.7% on average at 150 dpi (GLMM, df = 1, *z-value* = 6.003, *p*<0.0001; Figure 3A and Table S1). An analysis of four additional agro-inoculated plants in Exp. 3 showed that the proportion of recombinants remains high at a later stage of the co-infection, with 62% on average by 330 dpi (Figure 3A).

In Exp 4, the frequency of recombinant genomes was examined more intensively during the first 30 dpi. During this time-period, a real-time PCR-based test was used to detect the presence and the frequency of recombinant genomes in viral populations from 22 co-infected plants at 18 and 30 dpi (Figure 1). Recombinant genomes were detected in 5 out of 22 plants at 18 dpi with a maximum frequency of 0.1% (7.94 10^−4^±2.63 10^−4^) and in all plants at 30 dpi, comprising approximately 0.2% of the viral genomes detected at 30 dpi (17.4 10^−4^±13.2 10^−4^).

All previous studies estimating the frequencies of either cell co-infections or recombinant genomes during begomovirus mixed infections were performed on plants co-infected by co-agro-inoculation of parental viruses [13], [23]. However, co-infection of begomoviruses in nature, and the consequent generation of recombinant genomes, will depend on the capacity of co-inoculated viruses to meet within cells after vector transmission. This capacity was studied by comparing the frequencies of recombinant genomes present in viral populations detected from agro-inoculated plants with those detected in vector-inoculated plants. The viral populations within vector-inoculated plants were derived from two different agro-inoculated plants at 30 dpi as described above (source plants 1 and 4; Figure 1) with an individual female of *B. tabaci*. At the moment of vector transmission, RFLP analysis indicated that the viral population within source plant 1 was composed of 61% TYX and 39% TOX, and that in source plant 4 comprised 75.6% TYX, 22% TOX and 2.4% of recombinant genomes. Although all the whiteflies tested positive for both parental viruses, only three co-infected plants (labelled 1V1, 1V2 and 1V3) were obtained during transmissions from source plant 1, and two co-infected plants (labelled 4V1 and 4V2) were obtained following transmissions from source plant 4 (Figure 1). The proportions of recombinants within viral populations present in these five vector transmitted plants were around 4% at 60 dpi and 51% at 150 dpi (Figure 3B).

Comparing the proportion of recombinant genomes detected in agro-inoculated and vector-inoculated plants indicated that the inoculation method had no obvious significant effect on the accumulation of recombinants (GLMM, df = 1, *z-value* = –1.686, *p* = 0.092; Table 2). As in agro-inoculated plants, the frequency of recombinant genomes in vector-inoculated plants increased significantly with time between 60 and 150 dpi (GLMM, df = 1, *z–value* = 8.103, *p*<0.0001; Figure 3 and Table S2). However, the population-wide proportions of recombinant genomes increased more in vector-inoculated plants than they did in agro-inoculated plants as indicated by the significant interaction effect between time and inoculation method (GLMM, df = 1, *z-value* = 2.171, *p* = 0.030; Table 2).

**Table 2:** Effects of the co-inoculation method and the time after inoculation on the frequency of *Tomato yellow leaf curl virus* (TYX), *Tomato leaf curl Comoros vir*us (TOX) and recombinant genomes (REC) in co-infected tomato plants.

Genome	Factor	*z-value*	*p-value* 1
TYX	Inoculation method	−2.083	0.037*
	Time	−6.206	<0.0001***
	Time×inoculation method	0.952	0.34
TOX	Inoculation method	3.386	0.0007**
	Time	2.044	0.041*
	Time×inoculation method	−3.055	0.0023*
REC	Inoculation method	−1.686	0.092
	Time	5.633	<0.0001***
	Time×inoculation method	2.171	0.030*

Generalized linear mixed model, df = 1.

1 Significant effects (*) *P*<0.05. (**) *P*<0.001 and (***) *P*<0.0001.

### Frequency of Parental Genomes Over Time in Agro-inoculated and Vector-inoculated Plants

In the four agro-inoculated plants and in the five vector-inoculated plants examined during Exps. 1 and 2, TYX accounted for more genomes than TOX at both 30 and 60 dpi (Figure 3). Although the inoculation method has a marginal significant effect on the frequency of TYX (GLMM, df = 1, *z-value* = –2.083, *p* = 0.037; Table 2), the frequencies of TYX genomes decreased significantly between 60 and 150 dpi in both agro-inoculated and vector-inoculated plants (GLMM, *p*≤0.0235; Figure 3, Table S1 and Table S2). This decrease was confirmed by 330 dpi samples collected during Exp. 3 in agro-inoculated plants (Figure 3A).

The mean frequency of TOX estimated from all plants (agro- and vector- inoculated) was approximately stable during co-infection with 18.8% (95% CI: 14.8–23.2%) at 60 dpi and 17.2% (12.2–23.2%) at 150 dpi). In agro-inoculated plants, the mean frequency of TOX did not differ significantly between 30 and 150 dpi (GLMM, df = 1, *z-value* = –1.576, *p* = 0.115; Figure 3A and Table S1) although a significant decrease was observed between 30 and 60 dpi (GLMM, df = 1, *z-value* = –4.333; *p*<0.0001 Figure 3A and Table S1). A significant decrease was also observed in vector inoculated plants between 60 and 150 dpi (GLMM, df = 1, *z-value* = –2.291 *p* = 0.022; Figure 3B and Table S2). A strong interaction effect was detected between time and inoculation method (GLMM, *p* = 0.0023, *z-value* = –3.055; Table 2) probably due to the significant effect of the inoculation method on the frequency of TOX (GLMM, *p* = 0.0007, *z-value* = 3.386; Table 2).

The 22 coinfected plants examined during Exp. 4 were tested for the presence of TYX and TOX using real time PCR and the frequencies of TYX prior to 30 dpi were deduced from the viral accumulation values (Figure 1). TOX was detected in 14 plants out of 22 at 18 dpi and in all plants at 30 dpi, whereas all plants tested positive for TYX at both dates. TYX was dominant at the early stages of infection (mean frequency of 92% at 18 dpi and 69% and 30 dpi) similar to that observed at 30 dpi in Exps. 1 and 2 (Figure 3).

### Recombination Patterns

A total of 833 genomes were cloned and analysed from samples collected at 30, 60, 150, and 330 dpi as described in Figure 1; 177 clones (21.2%) were identified as recombinants by RFLP analyses and 96 of these were fully sequenced (2, 24, 54 and 16 clones from samplings of 30, 60, 150 and 330 dpi, respectively). The two parental genomes differed at 531 nucleotide positions spread along their 2.8-kb genomes; these nucleotide polymorphisms could be used to identify the approximate positions of recombination breakpoints within recombinant genomes.

A total of 48 distinct genotypes were identified from the sequence analysis of the 96 recombinants genotypes (Figure S1). Among these, 13 were sampled at least twice in the same plant. Moreover, four recombinant genotypes were isolated from at least two different plants, a likely signature of selection (Figure 1). For example, recombinant genotype R10 was detected in 150 dpi samples collected from six independently inoculated plants, three plants infected by agro-inoculation and three by vector transmission. Also, genotype R10 was sampled between two and six times in four of these six plants. Interestingly, R10 was not detected in any of the 60 dpi samples.

The proportion of genotypes detected multiple times in plants increased progressively with time (Table 3). We detected a total of 175 different recombination breakpoints spread along the genomes. The observed recombinant genotypes were highly diverse, with 2 to 28 breakpoints per genotype. Although the frequency of recombinant genomes increased significantly up to 150 dpi (Figure 3), the mean number of breakpoints per distinct genotype did not increase with time (Table 3). About half (68/118) of the breakpoints detected among the 150 dpi sequences were already present in the 60 dpi genotypes, which means that the other 50 breakpoints were detected for the first time at 150 dpi (Figure S2).

**Table 3:** Percentage of recombinant genotypes sampled multiple times on tomato plants co-infected with *Tomato yellow leaf curl virus* (TYX) and *Tomato leaf curl Comoros virus* (TOX) at each of the sampling days post inoculation (dpi).

dpi	Plants sampled	Sequencedrecombinants	Different genotypes	Multiple genotypes (%)	Average breaks per genotype 1
30	4	2	2	0 (0%)	6 (12/2)
60	9	24	18	2 (11%)	7 (126/18)
150	8	54	26	11 (42%)	4.5 (118/26)
330	2	16	7	6 (86%)	3.6 (25/7)

1 Within brackets: ratio of the total number of breakpoints in the distinct genotypes to the number of distinct genotypes.

**Figure 4:**
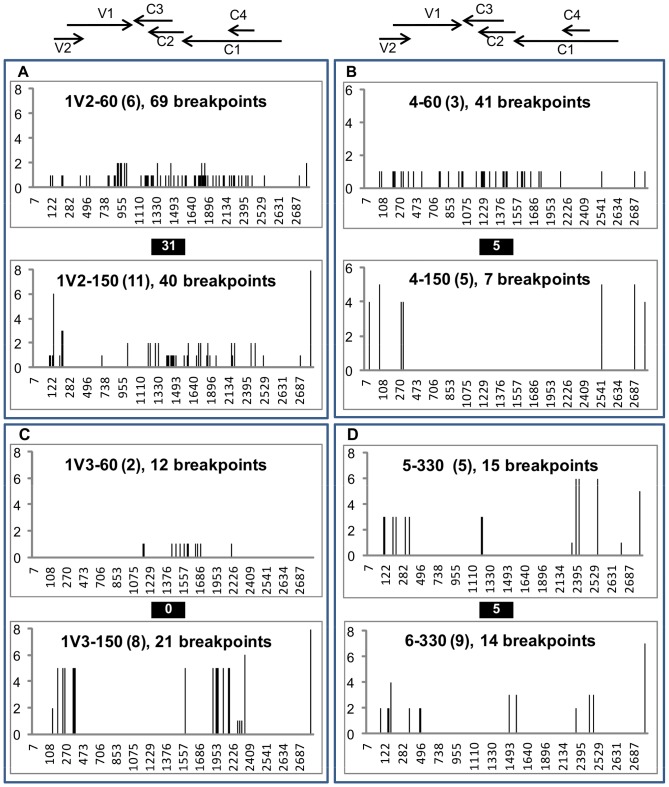
Distribution of recombination breakpoints detected in viral genomes isolated from tomato plants co-infected with TYX and TOX. Recombinant genomes were isolated from tomato plant co-infected with *Tomato yellow leaf curl virus* (TYX) and *Tomato leaf curl Comoros virus* (TOX) and sampled at 60, 150 and 330 days post inoculation (dpi) as reported in Figure 1. The distributions of recombination breakpoints are compared between genomes isolated at 60 dpi and 150 dpi from plants 4, 1V2 and 1V3 (**A, B, C**, respectively). Similarly, the distribution of breakpoints was compared between plants 5 and 6 at 330 dpi (**D)**. The breakpoints are presented on a genome linearized at the virion strand origin of replication and were located according to the nucleotide positions on the genome (x-axis). The positions of the six open reading frames (V1, V2, C1, C2, C3 and C4) are given at the top. The numbers in brackets indicate the numbers of genomes analyzed in each sample, followed by the number of distinct breakpoints among these genomes. The number of common breakpoints detected between recombinant genomes sampled from the same plant at two dates (**A-C**) or between two 330 dpi plants (**D**) is shown in black boxes. The y-axis indicates the number of genomes in which each breakpoint was detected.

Breakpoint patterns often differ markedly among samples collected both from the same plant at different dates and from different plants at the same date (Figure 4 and Figure S2). In some individual plants more than 50% of the breaks detected at 150 dpi were already detected at 60 dpi (e.g., plant 1V2 and 4, see Figure 4A and B), whereas in other plants none of them were observed at 60 dpi (e.g., plant 1V3, see Figures 4C, respectively). Breakpoint patterns also differed between the two plants at 330 dpi (Figure 4D).

### Infectivity of Six Recombinant Genomes in Tomato Plants

We have previously shown that the *in vitro* generated recombinants of TYX and TOX were all infectious [8]. However, the infectivity of recombinants generated *in vivo* has never been tested. We tested the infectivity of six recombinant genomes that had been detected multiple times in 330 dpi co-infected plants (Table S3). Each clone was agro-inoculated into 11 to 20 tomato plants and the percentage of infected plants was determined from real-time PCR tests at 30 dpi. They were all infectious, inducing typical TYX- or TOX-like symptoms, and their infectivities (45.5–89.5%) were similar to, or higher than, those of their parental genomes (TOX, 35% and TYX, 58.3%). Clone R4 induced the most severe symptoms of all the clones tested; these consisted of a dramatic reduction in plant growth, leaf distortion and yellowing of leaves and stems (Figure S3). It should be noted that the co-infected plant from which this clone was isolated at 330 dpi (plant 5, Figure 1) exhibited these same symptoms from 110 dpi onwards.

### Fitness of the Highly Virulent R4 Recombinant

Due to its high virulence, we analysed several fitness components of recombinant R4 to assess its emergence potential. More specifically, we compared R4 and parental viruses for infectivity and within-host viral load in co-inoculated tomato plants (competitiveness). The parental viruses and R4 were also compared for their efficiency in vector-transmission.

#### Competitiveness of recombinant R4 in mixed infection with the parental viruses in tomato

**Figure 5:**
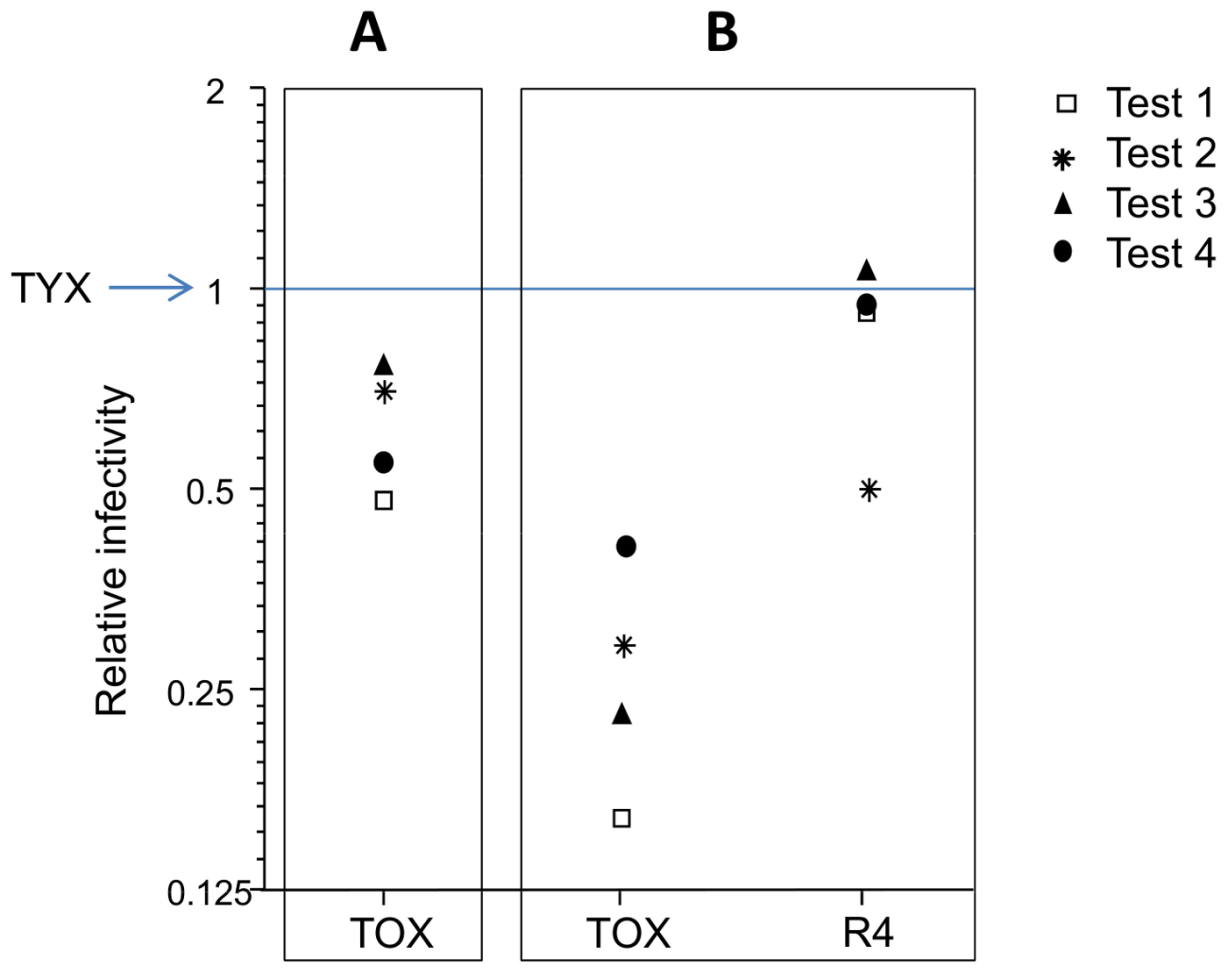
Relative infectivities of the recombinant R4 and the parental clones TYX and TOX in tomato. The infectivities were determined 30 days after co-inoculation of tomato plants with *Tomato yellow leaf curl virus* (TYX) and *Tomato leaf curl Comoros virus* (TOX) (A) or with TYX, TOX and recombinant R4 (B). Infectivity (number of infected plants/number of inoculated plants) was determined from four independent tests (1–4) comprising 30 to 45 plants each in (A) and 60 to 90 plants each in (B). In both graphs, the infectivity of TYX is arbitrarily set to 1 (blue line).

The competitiveness of recombinant R4 in mixed infections with the two parental viruses was estimated in four independent tests. Although the use of NZY+ medium (test 3) instead of LB medium (test 1, 2 and 4) has generally improved the efficiency of the agroinoculation of the three competitors, it did not substantially change the estimation of the relative infectivity of R4 at 30 dpi and its relative accumulation in plants at 18 and 30 dpi. It has to be noted that the newly generated recombinants inevitably generated during the competition test (see above), were expected to account for a maximum of, on average, 1% of all viral genomes (see above and Figures 1 and 3). Their influence on the competition test was expected to be negligible because of their low frequency and because of their high genetic variability (see section “Recombination pattern”) which is expected to result in a global null effect. In each of these four triple inoculation tests, a set of control plants was co-inoculated only with TYX and TOX and similarly monitored. In all the four control sets, fewer plants were detectably infected with TOX than were infected with TYX at 30 dpi (Figure 5A and Table S4). Moreover, a global analysis of the four control tests showed that the viral load of TYX genome was significantly higher than that of TOX (Figure S4A) at 18 dpi ANOVA, df = 1, F = 65.42, *p*<0.0001) and at 30 dpi (ANOVA, df = 1, F = 40.38, *p*<0.0001), as observed in Exp. 4 (Figure 1 and Figure S4A).

Analysis of infectivities in the TYX+TOX+R4 inoculated plants revealed that the frequency of TOX-infected plants was consistently lower than in the control TYX+TOX inoculation tests (Figure 5B) at 30 dpi. The infectivity of R4 was consistently higher than that of TOX and was similar to that of TYX. Thus, the infectivity of TOX seemed to be hindered substantially in the presence of R4 (Figure 5B). This may explain why the independence of infectivity between co-inoculated viruses was rejected (G2 statistic test, G2>14.26, *p*≤0.007) in three TYX+TOX+R4 tests whereas it was not in the TYX+TOX controls of each of these three tests (Fisher’s exact test, *p*≥0.25). Interestingly, the test that did not follow this independence pattern was the test in which NZY+ medium was used instead of LB medium.

The viral load was estimated only in plants co-infected with R4 and TYX, because too few plants were co-infected with TOX and R4 or with the three viruses. A global analysis of the four tests showed a significant dominance of TYX genome (Figure S4B) at 18 dpi (ANOVA, df = 1, F = 55.21, *p*<0.0001) and at 30 dpi (ANOVA, df = 1, F = 32.59, *p<*0.0001). Moreover, the relative frequency of R4 was not found to be significantly different between 18 and 30 dpi in tests 2 and 3 (MANOVA, *p*≥0.093).

#### Efficiency of vector transmission of TYX, TOX and recombinant R4 to tomato plants

Since it was unknown whether R4 was even transmissible by *B. tabaci*, we tested its transmission efficiency and compared it to that of TYX and TOX. Each viral clone was transmitted from three independent infected source plants. Virus accumulation was estimated in each of the source plants 2 days before the beginning of the acquisition period by real-time PCR. The plants infected with TYX and TOX contained approximately 6×10^6^ DNA copies/mg of leaf sample, whereas those infected by R4 contained 6×10^7^ copies/mg. The three genotypes were transmitted at different rates at 30 dpi (hierarchical GLM model, df = 2, χ2 = 21.17, *p<0*.0001). The transmission efficiency of R4 was 7.5% (95% CI: 2.8–14.1%) whereas the transmission efficiencies of TYX and TOX were estimated to be 24.5% (15.8–34.6%) and 3.24% (0.97–7.5%), respectively. The transmission efficiency of R4 was significantly lower than that of TYX (Fisher’s exact test; *p = *0.004) and marginally higher than that of TOX (Fisher’s exact test; *p = *0.067; Table S5).

### Estimation of Population Bottlenecks during Host Colonization by Begomoviruses

Stochastic phenomena during systemic infection could account for the observed differences in recombination patterns among plants at a given time point or within a plant at different time points. We estimated the magnitude of population bottlenecks that could induce such stochasticity during plant colonisation between 18 and 30 dpi using a dataset of the competition test. The data set of competition test 3 was used because this test was the only one exhibiting 3 features allowing for a robust estimation of the competitiveness and the bottleneck size: (i) initial frequency values were distributed over the whole range of the frequency spectrum (i.e. frequencies at 18 dpi were not all clustered around low values, Figure S5B), (ii) the number of test plants co-infected with TYX and R4 was the largest among tests, and (iii) changes in the relative frequencies of TYX and R4 were not found to differ significantly over the time interval of the experiment (Figure S5A and S5B). A severe population bottleneck was detected between the two dates such that only approximately 11 genomes (95% confidence interval: 3.5–320.5) from the 18-dpi population founded the population at 30 dpi.

## Discussion

Recombination is one of the main forces shaping virus evolution, sometimes leading to the emergence of new genotypes with increased host ranges or virulence. As a result, a large number of studies have analyzed, among other factors, the various constraints on either genetic exchange or the epidemiology of recombinants. However, the dynamics of recombinant generation and maintenance during infection of multicellular hosts has never been studied in detail. To our knowledge, the few available studies on the subject have focused either on a single infected individual or on a single time point, hampering efforts to disentangle the relative influences of selection and genetic drift on observed recombination patterns. Here, we analyzed in detail recombinational dynamics and the intensity of drift occurring during host colonization using a plant virus model. Besides the fundamental interest of the question, the results generated in this study may help to predict the outcome of mixed infections of TYLCV and ToLCKMV, the two begomoviruses used for this study, which will probably occur following likely contacts between these viruses in the SWIO at some time in the near future.

The analysis of viral populations within co-infected plants was based on the cost-effective approach of detecting recombinant clones for later sequencing. This approach involved an RCA-based amplification of virus genomes followed by cloning, detecting recombinant genomes by restriction analysis and full-genome sequencing. The RFLP analysis involved 17 restrictions sites situated all along the 2.8-kb genomes (Figure 2). We cloned and analyzed a total of 833 genomes with RFLP and fully sequenced almost 100 recombinant genomes. This relatively large sample allowed us to explore viral populations in depth, enabling us to observe signatures of both selection and drift (discussed below). We did not detect any recombinants after RFLP analysis of 174 genomes isolated from four agroinoculated plants (plants 1 and 3 at 30 dpi and plant 1V1 and 4V1 at 60 dpi (Figure 1), and the sequencing of 29 clones (15 TYX, 14 TOX) chosen randomly among those exhibiting parental RFLP profiles did not reveal any ‘hidden’ recombinants. This result confirmed that the polymerase used in the RCA is not inherently recombinogenic and, as previously observed [13], is therefore well suited for analyzing recombinants. This RCA/RFLP-based approach did, however, underestimate the numbers of recombinant genomes present in mixed infections because it could not detect recombination patterns in which recombinationally derived genome fragments were too small to contain any of the discriminating restriction sites. Consistently, we determined that 10 out of 50 distinct recombinants identified previously by full-length sequencing [15] would have been missed with our screening. The slight bias introduced by this underestimation of recombination breakpoints in certain genome regions implied that it was not valid to use the previously reported simulation based approaches to confirm the identified mechanistic and selective constraints on begomovirus recombination shown previously in [15].

### Dynamics of Recombinant Genome Accumulation and the Effect of Natural Vector Transmission

Recombination, and thus co-infection, took place early during infection in our model. Recombinants of TYX and TOX were cloned from plant samples collected as early as 30 dpi–much earlier than the detection of begomovirus recombinants at ∼120 dpi in some other similar experiments [13], [15]. The earliest time point at which recombinant genomes can be detected is obviously dependent on the sampling effort. As a result of analysing a relatively greater number of genomes than in many previous studies, we were able to detect a very low frequency of recombinants at 30 dpi (≈ 1%). This early detection of recombinants was confirmed using a complementary real-time PCR approach, which showed that recombinants were indeed detectable at 30 dpi and even at 18 dpi in some plants. These results indicate that cell co-infection can take place early in infection, as previously shown in co-infections of TYLCV and the related begomovirus TYLCSV [23]. The frequency of recombinants increased progressively with a moderate frequency at 60 dpi (≈ 10%) and a significantly higher frequency at 150 dpi (≈50%). After this time point, the frequency of recombinant genomes did not increase much, remaining in much the same range as frequencies previously seen at similar time-points in other begomovirus co-infection studies [13], [15].

It is possible that the artificial co-infection method based on using *A. tumefaciens* may have induced a rate of cell co-infection, and a frequency of recombinant genomes, higher than that which would have been obtained with natural infection of plants using the whitefly vector. To test this possible bias, we compared co-infected plants inoculated either artificially or naturally. Interestingly, irrespective of the virus inoculation method, similar recombinant genome accumulation dynamics were observed, validating the use of agro-inoculation to simulate natural co-infections and study recombination among begomoviruses.

### Stochastic Effects and Natural Selection Acting on Population Dynamics

Here, we estimated quantitatively for the first time the magnitude of population bottlenecks encountered by begomovirus populations during host colonization. The low numbers of genomes present at 18 dpi that were required to found the virus population at 30 dpi (11 genomes ±3.5–320.5; median ±95% CI) suggests that genetic drift can be rather intense during the initial phases of systemic infection. Within-host population bottlenecks have been quantified only rarely in viruses. In plant viruses, most estimates come from RNA viruses and also indicate severe bottlenecks ([24]–[27]. The only counter example (i.e. a large colonizing population) comes from a double-stranded DNA virus [22], [28]. These observations, among others, have led to models of within-host evolution of plant RNA virus in which genetic drift is a major process [25]. Our data suggest that single-stranded DNA viruses can also encounter intense drift during plant infection.

The patterns and dynamics of the recombination breakpoints observed between 60 dpi and 150 dpi may also be informative on this particular point. The recombinant genomes isolated at those two time points were highly diverse among plants. Moreover, the breakpoint distributions detected at 150 dpi were not predictable from the breakpoint distributions detected at 60 dpi in the same plants (Figure 4, Figure S2). The fact that within the same plant recombination breakpoints observed at 60 dpi were not observed at 150 dpi is consistent with severe bottlenecks taking place even after 30 dpi. From another perspective, the fact that in each of the plants, breakpoints detected at 150 dpi were not seen at 60 dpi is consistent with recombinant genomes being continuously generated suggesting that co-infection of individual cells with genetically distinct viruses probably takes place throughout infections. A sampling bias in the case of a very high diversity of recombinant genomes in each plant would also explain the difference of recombination profiles between 60 and 150 dpi. This interpretation is however not consistent with the fact that many cases particular virus genotypes were sampled multiple times from individual plants (Table 3).

Despite apparently continuous generation of new recombinants and genetic drift, some data suggested that selection also acts on the viral populations. Each viral population in the infected plants can be considered as a lineage evolving in parallel since the viral genetic diversity within the initial inoculum populations was identical for all plants. Identical recombinant genotypes observed simultaneously in parallel lineages are therefore likely to have been selected for. In this study, four recombinant genomes were each isolated from several plants in parallel and from unrelated viral populations (i.e. populations that were not derived from one another by vector transmission, Figure 1). Interestingly, two of these recombinants (R10 and R6–22) are very similar to one of the recombinant genomes that was previously found at a high frequency in a TYX+TOX co-infection [15]. Other recombinant genomes isolated multiple times, but only from individual plants, may also have been selected for, but it cannot be excluded that these may also have risen to high frequencies by drift.

### Potential Outcomes of Natural Encounters between TYLCV and ToLCKMV

TYLCV is considered an important threat to tomato production. This virus has spread to several regions of the world including the islands of Mauritius and Réunion in the SWIO. The risk of contact between TYLCV and tomato begomoviruses such as ToLCKMV, which is indigenous to neighboring SWIO islands, is high [29]. As the recombinants detected in an artificially reconstituted TYLCV-TYLCSV mixed infection [11], [13] resembled those found in natural infections, it may be possible to predict the potential outcome of an encounter between TYLCV and ToLCKMV. According to previous experiments [15], some of the recombinant genomes isolated from plants co-infected with TYLCV and ToLCKMV were detected multiple times and were therefore supposed to be reasonably fit. These authors confirm the effect of selection by showing a non-random distribution of the recombination breakpoints which tended to preserve various categories of intra-genome interaction. In the present study, the effect of selection was further confirmed with recombinant genotypes detected multiple times. Most importantly, four of these recombinant genotypes were detected in multiple independent infections (Figure 1, ▪, ▴, □, R10). The fact that recombinant R10 was detected in six of thirteen test plants suggests that this recombinant genome has a high probability of being generated in tomato plants that become naturally infected with TYLCV and ToLCKMV. The only gene which was previously reported to determine vector transmission specificity in geminiviruses is the CP gene [30]. As the CP of R10 was derived from TYLCV, the most efficiently transmitted parent, R10 is expected to be easily transmitted between plants. Further studies are needed to confirm the emergence potential of R10 because the high adaptation of R10 to tomato detected in our experiments does not necessarily mean that it would be highly adapted to other host species. As observed with the highly virulent recombinant R4, some of the recombinants detected multiple times can be more virulent than both parental viruses. Recombinant R4 may be a potential threat under natural conditions because its within-host accumulation, infectivity and vector transmissibility were similar to those of the parental clones. However, as with R10, the emergence potential of R4 has to be further tested because of the complex selective processes occurring in nature where viruses must generally retain the capacity to infect a multitude of different host species and/or genotypes of these species. It is noteworthy that the three to four month duration of a tomato crop on the islands of the SWIO is, according to our results, compatible with the emergence of recombinant genomes within tomato plants (Figure 3). This period is also compatible with the maintenance of parental genomes which both constitute a relatively high proportion of the viral population at 150 dpi whatever the nature and the frequencies of recombinant genomes detected. Overall, these results represent a further step in our understanding of the potential events that would occur following a natural encounter between TYLCV and ToLCKMV.

## Supporting Information

Figure S1 **Recombinant genotypes isolated from tomato plants co-infected with TYX and TOX.** Recombinant genomes were isolated from tomato plant co-infected with *Tomato yellow leaf curl virus* (TYX) and *Tomato leaf curl Comoros virus* (TOX) as reported in Figure 1. All recombinant genomes that were fully sequenced are presented. Each line represents one recombinant linearized at the virion strand origin of replication; red fragments were derived from TYX and blue fragments from TOX. The positions of the six open reading frames (V1, V2, C1, C2, C3 and C4) are given at the top, and dashed lines indicate the position on the genome of all the 500 nucleotides. The recombinants are grouped in blocks according to the plant sample from which they were isolated. The plant samples are identified on the left side as follows: plant number (“V” indicates the plants which were co-infected by vector-inoculation) –sampling day after inoculation. When a recombinant was detected more than once in a sample, the number of times it was detected is indicated on the right side; the multiple recombinants that were tested for their infectivity (Table 3) are followed by a code number. The recombinant genomes that were isolated from at least 2 different samples are indicated as follow: •, *, ▴, □, R10, R6–22.
(TIF)

Figure S2 **Distribution of breakpoints along the recombinant genomes isolated from tomato plants co-infected with TYX and TOX.** Recombinant genomes were isolated from tomato plants co-infected with *Tomato yellow leaf curl virus* (TYX) and *Tomato leaf curl Comoros virus* (TOX) and sampled at 30, 60, 150 and 330 days post inoculation (dpi) as reported in Figure 1. The breakpoint positions are presented on a genome linearized at the virion strand origin of replication and were located according to the nucleotide positions (x-axis). The numbers in brackets indicate the total numbers of fully sequenced recombinant genomes that were isolated at each sampling date. The y-axis indicates the number of these genomes in which each breakpoint was detected. Red bars under the x-axis at 60 and at 150 dpi represented the breakpoints that were already detected on the previous sampling date. The positions of the six open reading frames (V1, V2, C1, C2, C3 and C4) are given at the top.
(TIF)

Figure S3 **Symptoms caused by parental and recombinant viral clones on tomato plants 40 days after inoculation.** Tomato plants of the cultivar Monalbo agro-infected with (from left to right) *Tomato leaf curl Comoros virus* (TOX), recombinant R10, recombinant R4, and *Tomato yellow leaf curl viru*s (TYX). A healthy control is shown on the right.
(TIF)

Figure S4 **Viral accumulation of TYX, TOX and R4 genomes within tomato plants co-infected with TYX and TOX or with TYX and R4.** A) A boxplot representation was used to show the distribution of the N0 values representing the virus accumulation at 18 and 30 dpi, in 47 plants coinfected with *Tomato yellow leaf curl virus* (TYX) and *Tomato leaf curl Comoros vir*us (TOX) from tests 1 to 4: within the boxes, the horizontal line indicates the median value (50% quantile); the box itself delimits the 25% and 75% quantiles, and the whiskers extend from the box indicate the lowest and the highest observed values. B) Distribution of the N0 values at 18 and 30 dpi, in 60 TYX-R4 coinfected tomato plants from tests 1 to 4.
(TIF)

Figure S5 **Frequency of R4 genomes within tomato plants co-infected with TYX.** A total of 22 tomato plants of the competition test 3 were detected to be co-infected with *Tomato yellow leaf curl virus* (TYX) and recombinant R4 at 18 and 30 days after inoculation (dpi). A) A boxplot representation was used to show the distribution of the frequency of R4 in the coinfected plants: within the boxes, the horizontal line indicates the median value (50% quantile); the box itself delimits the 25% and 75% quantiles, and the whiskers extend from the box to the lowest and highest observed value. B) Dynamics of the frequency of R4 in each of the 22 coinfected plant between 18 and 30 dpi.
(TIF)

Table S1 **Time effect on the frequency of** ***Tomato yellow leaf curl viru*** **s (TYX),** ***Tomato leaf curl Comoros virus*** **(TOX) and recombinant genomes in agro-inoculated tomato plants.** Generalised linear mixed model, df = 1.^ 1^ Significant effects: (*) *P*<0.05. (**) *P*<0.001 and (***) *P*<0.0001.
(DOCX)

Table S2 **Time effect on the frequency of** ***Tomato yellow leaf curl virus*** **(TYX),** ***Tomato leaf curl Comoros virus*** **(TOX) and recombinant genomes (REC) in vector-inoculated tomato plants.** Generalised linear mixed model, df = 1.^ 1^ Significant effects: (*) *P*<0.05. (**) *P*<0.001 and (***) *P*<0.0001
(DOCX)

Table S3 **Infectivity in tomato of** ***Tomato yellow leaf curl virus*** **(TYX),** ***Tomato leaf curl Comoros virus*** **(TOX) and six recombinant genomes.**
(DOCX)

Table S4 **Infectivity of** ***Tomato yellow leaf curl virus*** **(TYX),** ***Tomato leaf curl Comoros virus*** **(TOX) and recombinant R4 in competition tests.** Four competition tests are presented (Tests 1–4). In each set of competition tests a set of control plants was co-inoculated only with TYX and TOX. Infectivity was determined at 18 and 30 days post inoculation (dpi) for each viral clone, as the number of detectably infected plants relative to the number of inoculated plants.
*: Agroinfectious clones were cultured in LB medium except in Test 3 where NZY+ medium was used.
(DOCX)

Table S5 **Comparison of the transmission efficiency of** ***Tomato yellow leaf curl virus*** **(TYX) and** ***Tomato leaf curl Comoros virus*** **(TOX) and recombinant R4 between tomato plants using the natural vector** ***Bemisia tabaci*** **.** Transmission efficiency was determined as the ratio between infected and inoculated plants.
(DOCX)
