# Supplementary figures and images for: Within-Host Dynamics of the Emergence of Tomato Yellow Leaf Curl Virus Recombinants
Source: PLoS One. 2013 Mar 5;8(3):e58375. doi: 10.1371/journal.pone.0058375 (PMC3589402; doi:10.1371/journal.pone.0058375)

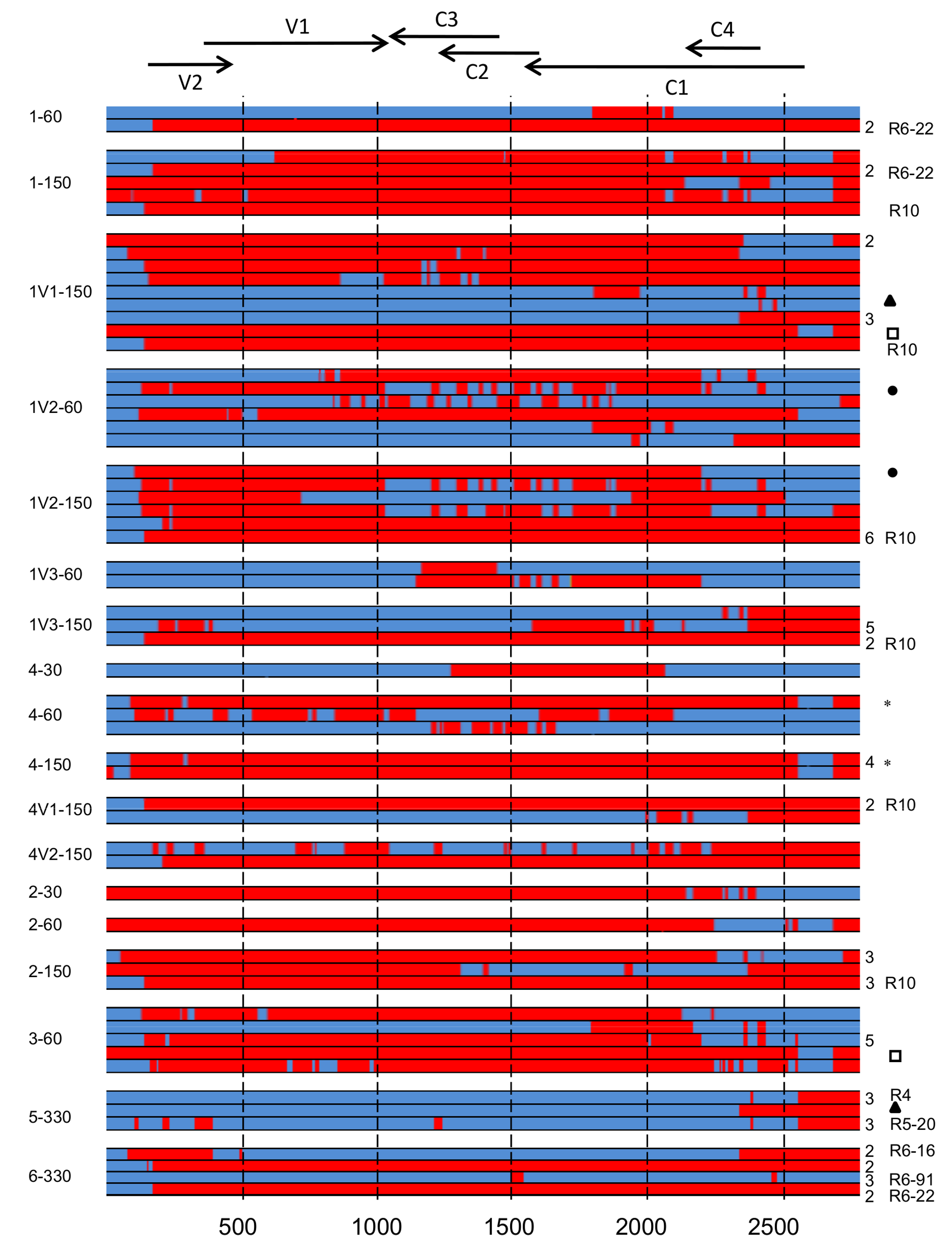

Supplement: Figure S1 — Recombinant genotypes isolated from tomato plants co-infected with TYX and TOX. Recombinant genomes were isolated from tomato plant co-infected with Tomato yellow leaf curl virus (TYX) and Tomato leaf curl Comoros virus (TOX) as reported in Figure 1. All recombinant genomes that were fully sequenced are presented. Each line represents one recombinant linearized at the virion strand origin of replication; red fragments were derived from TYX and blue fragments from TOX. The positions of the six open reading frames (V1, V2, C1, C2, C3 and C4) are given at the top, and dashed lines indicate the position on the genome of all the 500 nucleotides. The recombinants are grouped in blocks according to the plant sample from which they were isolated. The plant samples are identified on the left side as follows: plant number (“V” indicates the plants which were co-infected by vector-inoculation) –sampling day after inoculation. When a recombinant was detected more than once in a sample, the number of times it was detected is indicated on the right side; the multiple recombinants that were tested for their infectivity (Table 3) are followed by a code number. The recombinant genomes that were isolated from at least 2 different samples are indicated as follow: •, *, ▴, □, R10, R6–22. (TIF) [file pone.0058375.s001.tif]

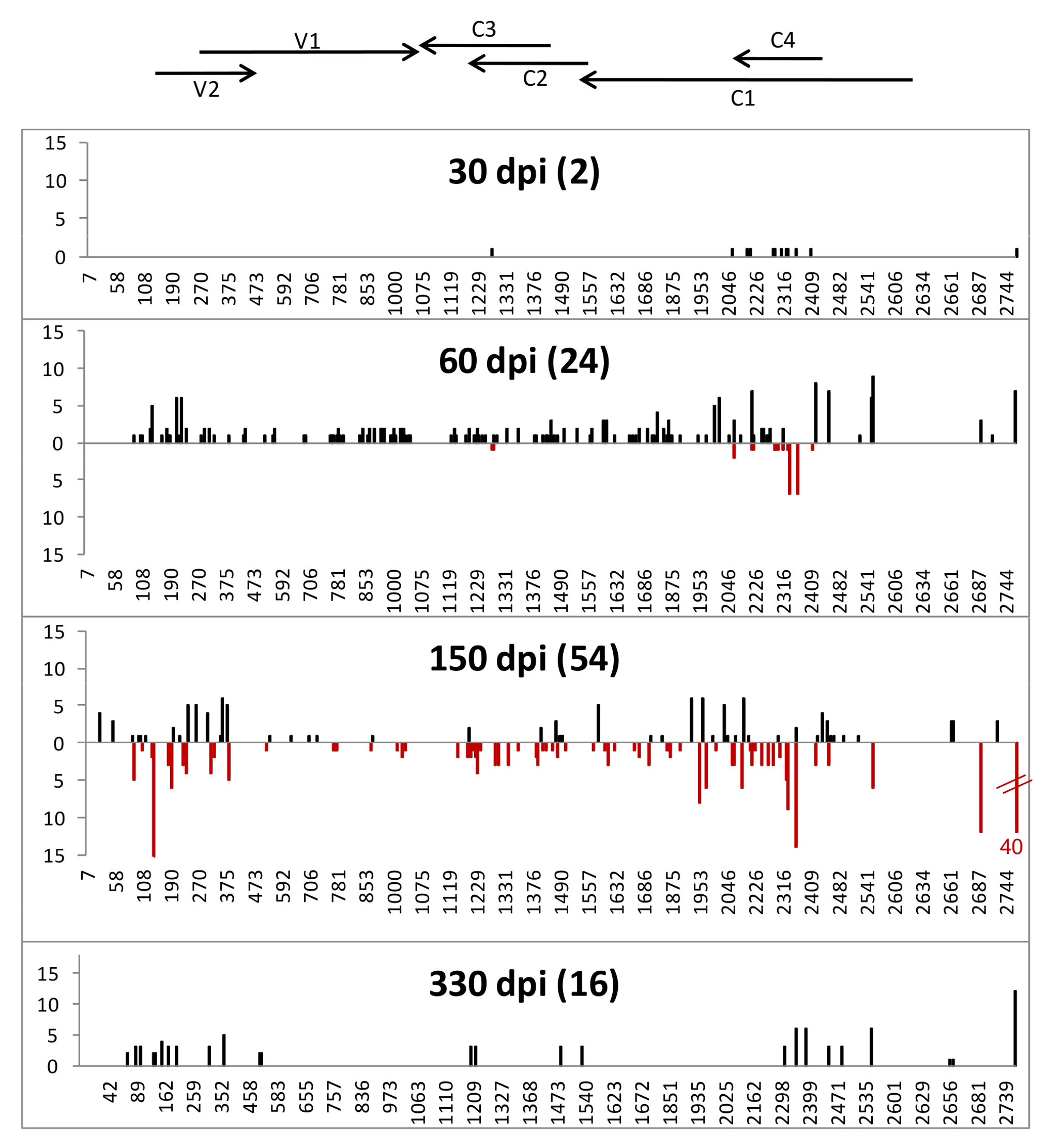

Supplement: Figure S2 — Distribution of breakpoints along the recombinant genomes isolated from tomato plants co-infected with TYX and TOX. Recombinant genomes were isolated from tomato plants co-infected with Tomato yellow leaf curl virus (TYX) and Tomato leaf curl Comoros virus (TOX) and sampled at 30, 60, 150 and 330 days post inoculation (dpi) as reported in Figure 1. The breakpoint positions are presented on a genome linearized at the virion strand origin of replication and were located according to the nucleotide positions (x-axis). The numbers in brackets indicate the total numbers of fully sequenced recombinant genomes that were isolated at each sampling date. The y-axis indicates the number of these genomes in which each breakpoint was detected. Red bars under the x-axis at 60 and at 150 dpi represented the breakpoints that were already detected on the previous sampling date. The positions of the six open reading frames (V1, V2, C1, C2, C3 and C4) are given at the top. (TIF) [file pone.0058375.s002.tif]

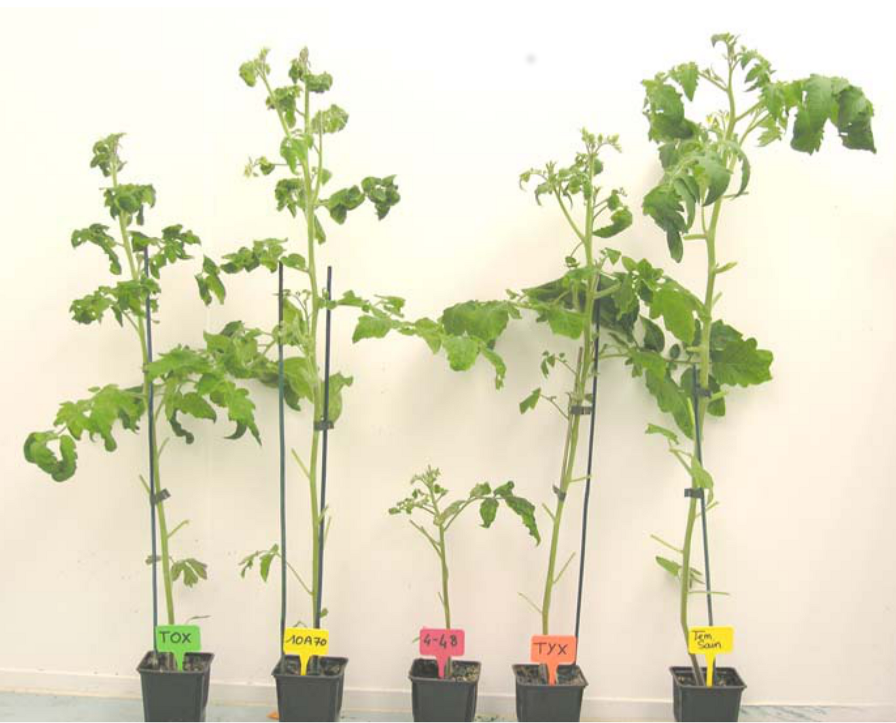

Supplement: Figure S3 — Symptoms caused by parental and recombinant viral clones on tomato plants 40 days after inoculation. Tomato plants of the cultivar Monalbo agro-infected with (from left to right) Tomato leaf curl Comoros virus (TOX), recombinant R10, recombinant R4, and Tomato yellow leaf curl virus (TYX). A healthy control is shown on the right. (TIF) [file pone.0058375.s003.tif]

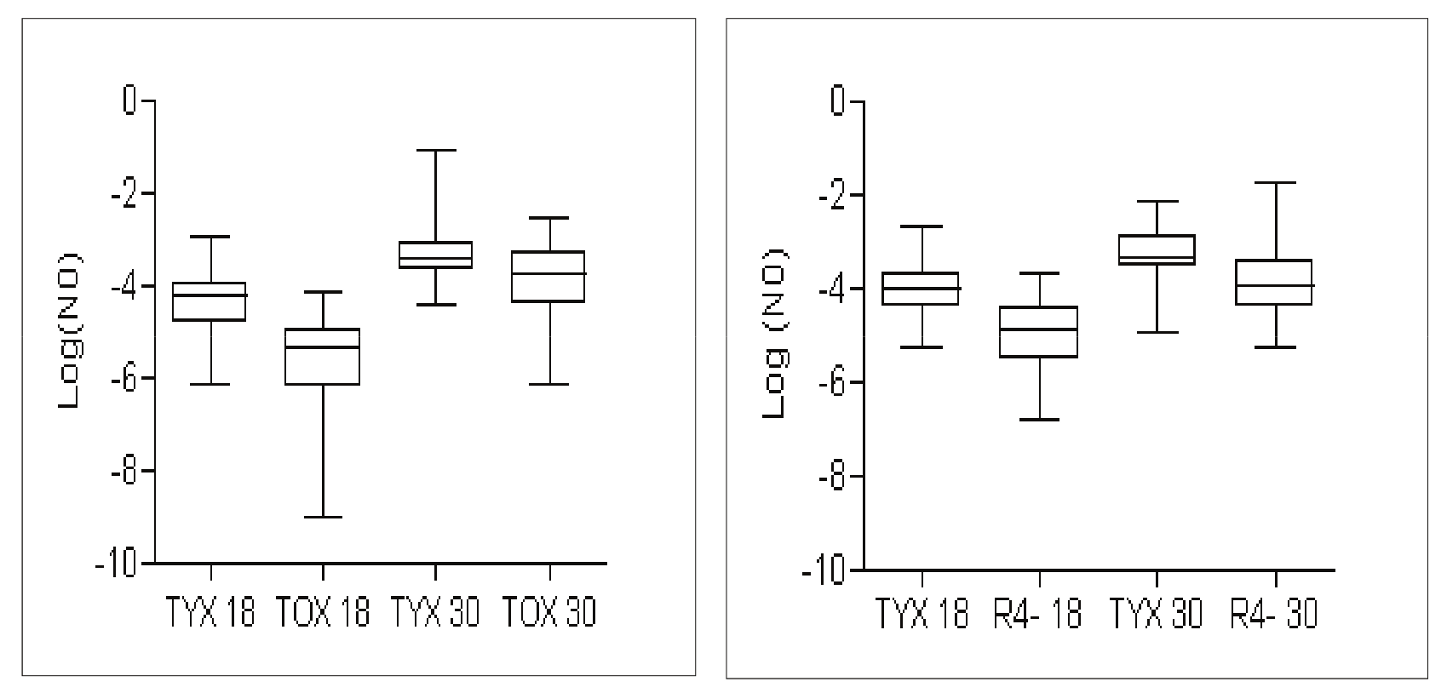

Supplement: Figure S4 — Viral accumulation of TYX, TOX and R4 genomes within tomato plants co-infected with TYX and TOX or with TYX and R4. A) A boxplot representation was used to show the distribution of the N0 values representing the virus accumulation at 18 and 30 dpi, in 47 plants coinfected with Tomato yellow leaf curl virus (TYX) and Tomato leaf curl Comoros virus (TOX) from tests 1 to 4: within the boxes, the horizontal line indicates the median value (50% quantile); the box itself delimits the 25% and 75% quantiles, and the whiskers extend from the box indicate the lowest and the highest observed values. B) Distribution of the N0 values at 18 and 30 dpi, in 60 TYX-R4 coinfected tomato plants from tests 1 to 4. (TIF) [file pone.0058375.s004.tif]

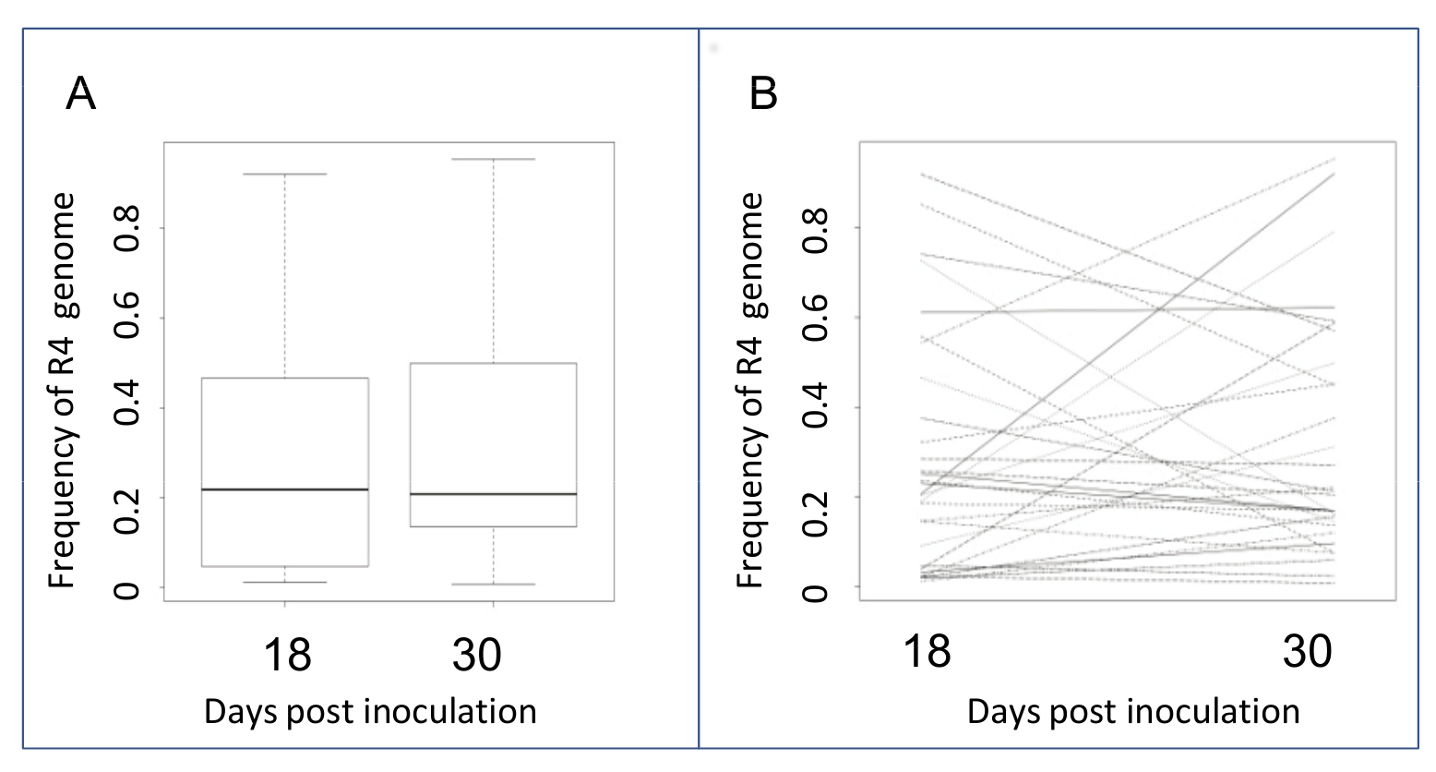

Supplement: Figure S5 — Frequency of R4 genomes within tomato plants co-infected with TYX. A total of 22 tomato plants of the competition test 3 were detected to be co-infected with Tomato yellow leaf curl virus (TYX) and recombinant R4 at 18 and 30 days after inoculation (dpi). A) A boxplot representation was used to show the distribution of the frequency of R4 in the coinfected plants: within the boxes, the horizontal line indicates the median value (50% quantile); the box itself delimits the 25% and 75% quantiles, and the whiskers extend from the box to the lowest and highest observed value. B) Dynamics of the frequency of R4 in each of the 22 coinfected plant between 18 and 30 dpi. (TIF) [file pone.0058375.s005.tif]
