# Supplementary material for: Within-Host Dynamics of the Emergence of Tomato Yellow Leaf Curl Virus Recombinants
Source: PLoS One. 2013 Mar 5;8(3):e58375. doi: 10.1371/journal.pone.0058375 (PMC3589402; doi:10.1371/journal.pone.0058375)
Supplement: Table S1 — Time effect on the frequency of Tomato yellow leaf curl viru s (TYX), Tomato leaf curl Comoros virus (TOX) and recombinant genomes in agro-inoculated tomato plants. Generalised linear mixed model, df = 1. 1 Significant effects: (*) P<0.05. (**) P<0.001 and (***) P<0.0001. (DOCX) [file pone.0058375.s006.docx]

| Genome | Factor | *z-value* | *p*^1^ |
| --- | --- | --- | --- |
| TYX | 30 dpi (intercept) | 4.294 | <0.0001*** |
|  | 60 dpi | 2.265 | 0.0235* |
|  | 150 dpi | -5.143 | <0.0001*** |
| TOX | 30 dpi (intercept) | -4.274 | <0.0001*** |
|  | 60 dpi | -4.333 | <0.0001*** |
|  | 150 dpi | -1.576 | 0.115 |
| REC | 30 dpi (intercept) | -5.950 | <0.0001*** |
|  | 60 dpi | 3.019 | 0.0025* |
|  | 150 dpi | 6.003 | <0.0001*** |
