# Supplementary material for: Within-Host Dynamics of the Emergence of Tomato Yellow Leaf Curl Virus Recombinants
Source: PLoS One. 2013 Mar 5;8(3):e58375. doi: 10.1371/journal.pone.0058375 (PMC3589402; doi:10.1371/journal.pone.0058375)
Supplement: Table S2 — Time effect on the frequency of Tomato yellow leaf curl virus (TYX), Tomato leaf curl Comoros virus (TOX) and recombinant genomes (REC) in vector-inoculated tomato plants. Generalised linear mixed model, df = 1. 1 Significant effects: (*) P<0.05. (**) P<0.001 and (***) P<0.0001 (DOCX) [file pone.0058375.s007.docx]

| Genome | Factor | *z-value* | *P* ^1^ |
| --- | --- | --- | --- |
| TYX | 60 dpi(intercept) | 3.027 | 0.00247 * |
|  | 150 dpi | -6.192 | <0.0001*** |
| TOX | 60 dpi (intercept) | -6.088 | <0.0001*** |
|  | 150 dpi | -2.291 | 0.022* |
| REC | 60 dpi (intercept) | -6.828 | <0.0001*** |
|  | 150 dpi | 8.103 | <0.0001*** |
