# Supplementary material for: Within-Host Dynamics of the Emergence of Tomato Yellow Leaf Curl Virus Recombinants
Source: PLoS One. 2013 Mar 5;8(3):e58375. doi: 10.1371/journal.pone.0058375 (PMC3589402; doi:10.1371/journal.pone.0058375)
Supplement: Table S3 — Infectivity in tomato of Tomato yellow leaf curl virus (TYX), Tomato leaf curl Comoros virus (TOX) and six recombinant genomes. (DOCX) [file pone.0058375.s008.docx]

| Recombinant clone (plant sample) | Number of agroinoculated plants | % infected plants |
| --- | --- | --- |
| R4 (5-330) | 19 | 89.5 |
| R6-91 (6-330) | 18 | 83.3 |
| R6-16 (6-330) | 13 | 76.9 |
| R10 | 20 | 75.0 |
| R6-22 (6-330) | 15 | 73.3 |
| **Tyx** | **12** | **58.3** |
| R5-20 (5-330) | 11 | 45.5 |
| **Tox** | **20** | **35.0** |
