# Supplementary material for: Within-Host Dynamics of the Emergence of Tomato Yellow Leaf Curl Virus Recombinants
Source: PLoS One. 2013 Mar 5;8(3):e58375. doi: 10.1371/journal.pone.0058375 (PMC3589402; doi:10.1371/journal.pone.0058375)
Supplement: Table S4 — Infectivity of Tomato yellow leaf curl virus (TYX), Tomato leaf curl Comoros virus (TOX) and recombinant R4 in competition tests. Four competition tests are presented (Tests 1–4). In each set of competition tests a set of control plants was co-inoculated only with TYX and TOX. Infectivity was determined at 18 and 30 days post inoculation (dpi) for each viral clone, as the number of detectably infected plants relative to the number of inoculated plants. *: Agroinfectious clones were cultured in LB medium except in Test 3 where NZY+ medium was used. (DOCX) [file pone.0058375.s009.docx]

| TYX+ TOX+ R4 (18) | number of plant | % infected | TYX(%) | TOX (%) | R4 (%) |
| --- | --- | --- | --- | --- | --- |
| Test 1 | 90 | 46,67 | 38,89 | 5,56 | 36,67 |
| Test 2 | 60 | 43,33 | 35,00 | 3,33 | 20,00 |
| Test 3***** | 60 | 88,33 | 66,67 | 8,33 | 68,33 |
| Test 4 | 90 | 74,44 | 37,78 | 17,78 | 57,78 |
| TYX+ TOX+ R4 (30) |  |  |  |  |  |
| Test 1 | 90 | 50,00 | 42,22 | 6,67 | 38,89 |
| Test 2 | 60 | 46,67 | 40,00 | 11,67 | 20,00 |
| Test 3***** | 60 | 95,00 | 71,67 | 16,67 | 76,67 |
| Test 4 | 90 | 83,33 | 71,11 | 28,89 | 66,67 |
| TYX+ TOX (18) |  |  |  |  |  |
| Test 1 | 35 | 42,86 | 42,86 | 11,43 |  |
| Test 2 | 30 | 66,67 | 63,33 | 33,33 |  |
| Test 3***** | 30 | 100,00 | 96,67 | 46,67 |  |
| Test 4 | 45 | 71,11 | 66,67 | 22,22 |  |
| TYX+ TOX (30) |  |  |  |  |  |
| Test 1 | 35 | 65,71 | 60,00 | 28,57 |  |
| Test 2 | 30 | 66,67 | 66,67 | 46,67 |  |
| Test 3***** | 30 | 100,00 | 100,00 | 76,67 |  |
| Test 4 | 45 | 82,22 | 75,56 | 42,22 |  |
