# Supplementary material for: Within-Host Dynamics of the Emergence of Tomato Yellow Leaf Curl Virus Recombinants
Source: PLoS One. 2013 Mar 5;8(3):e58375. doi: 10.1371/journal.pone.0058375 (PMC3589402; doi:10.1371/journal.pone.0058375)
Supplement: Table S5 — Comparison of the transmission efficiency of Tomato yellow leaf curl virus (TYX) and Tomato leaf curl Comoros virus (TOX) and recombinant R4 between tomato plants using the natural vector Bemisia tabaci . Transmission efficiency was determined as the ratio between infected and inoculated plants. (DOCX) [file pone.0058375.s010.docx]

| Source plants | TYX | TOX | R4 |
| --- | --- | --- | --- |
| Source plant 1 | 10/28 | 2/40 | 3/39 |
| Source plant 2 | 8/30 | 1/39 | 1/40 |
| Source plant 3 | 4/30 | 1/39 | 5/39 |
| Mean transmission rate | 24.5% | 3.24% | 7.5% |
